# Information Theory and Coding for Image and Video Processing

**DOI:** 10.3390/e28030294

**Published:** 2026-03-05

**Authors:** Ofer Hadar

**Affiliations:** School of Electrical and Computer Engineering, Ben-Gurion University of the Negev, Beer-Sheva 8410501, Israel; hadar@bgu.ac.il

## 1. Introduction and Scope of the Special Issue

Recent advances in image and video processing have been profoundly influenced by developments in information theory, source coding, and learning-based representations. Classical concepts such as entropy modeling, rate-distortion optimization, transform coding, and quantization are now being revisited in the context of deep learning, perceptual optimization, and large-scale multimedia systems.

This Special Issue, “Information Theory and Coding for Image and Video Processing”, aimed to bring together high-quality contributions that explore both theoretical foundations and practical coding methods for image and video data. Particular emphasis was placed on approaches that combine information-theoretic principles with modern signal processing and machine learning techniques.

Following a rigorous peer-review process, this Special Issue includes eight accepted articles, covering topics ranging from image and video compression, transform coding, and quantization, to adaptive metrics, perceptual optimization, and survey-level analyses of emerging trends.

## 2. Survey and Review Contributions

A comprehensive survey by Huang and Wu reviews recent advances in end-to-end learned image compression, offering a unified view of how classical rate-distortion theory and entropy modeling are integrated within modern deep learning frameworks [contribution 8]. The paper provides an extensive discussion of both human- and machine-oriented coding paradigms and outlines key challenges for future research.

Complementing this survey, Podgorelec et al. analyze state-of-the-art trends in data compression through the COMPROMISE case study, emphasizing practical system-level considerations and benchmarking methodologies [contribution 3]. Their work highlights how theoretical advances translate into real-world compression performance.

## 3. Image Compression and Transform Coding Methods

Several contributions focus on image compression techniques grounded in transform coding and dimensionality reduction. Ranjan and Kumar propose an improved image compression algorithm that combines two-dimensional discrete wavelet transforms with principal component analysis and canonical Huffman encoding, demonstrating enhanced compression efficiency while preserving image quality [contribution 7].

Žalik et al. introduce a novel transformation technique aimed at reducing information entropy in greyscale raster images, offering insights into redundancy reduction and entropy minimization from a structural perspective [contribution 6]. Their results reinforce the relevance of information-theoretic optimization in image representation.

In a learning-based direction, Gong et al. propose a convolutional neural network–based quantization method for block compressed sensing of images, integrating deep feature learning with quantization design to improve reconstruction performance [contribution 4].

## 4. Video Coding and Perceptual Optimization

Video compression and perceptual quality optimization are addressed in several papers. Shao et al. introduce a two-stage learning-based video compression framework that leverages hierarchical representations to achieve improved coding efficiency and reconstruction quality [contribution 2].

In the context of remote driving and teleoperation, Dror and Hadar investigate perceptually optimized video compression tailored to traffic signs and lights, employing multi-category region-of-interest coding to enhance visual fidelity in safety-critical regions [contribution 1]. This work demonstrates the importance of task-driven and perception-aware coding strategies for autonomous systems.

## 5. Adaptive Metrics and System-Level Optimization

Beyond core compression algorithms, adaptive metrics and system-level considerations are explored by Wang and Lu, who propose influential metric estimation and dynamic frequency selection mechanisms for JPEG-reversible data hiding [contribution 5]. Their two-dimensional mapping approach provides valuable insights into adaptive optimization under system constraints and highlights the interplay between coding efficiency and auxiliary objectives.

## 6. Concluding Remarks

The contributions collected in this Special Issue demonstrate that information theory remains a cornerstone of modern image and video processing research. From classical transform coding and entropy reduction techniques [contribution 6,7] to deep learning-based compression and quantization methods [contribution 2,4,8], and from perceptually optimized video coding [contribution 1] to adaptive system-level strategies [contribution 5], the papers collectively highlight the breadth and vitality of this research area.

We hope that this Special Issue serves as a useful reference for researchers and practitioners working at the intersection of information theory, signal processing, and multimedia systems, and will stimulate further advances in efficient, interpretable, and perceptually aware visual coding methodologies.

